# Validation of the functional assessment of cancer therapy-gastric module for the Chinese population

**DOI:** 10.1186/1477-7525-10-145

**Published:** 2012-11-30

**Authors:** Hui Jun Zhou, Jimmy BY So, Wei Peng Yong, Nan Luo, Feng Zhu, Nasheen Naidoo, Shu Chuen Li, Khay Guan Yeoh

**Affiliations:** 1 Saw Swee Hock School of Public Health, National University of Singapore, 16 Medical Drive, Block MD3, Singapore 117597, Singapore; 2 Department of Surgery, Yong Loo Lin School of Medicine, National University of Singapore, NUHS Tower Block, Level 8, 1E Kent Ridge Road, Singapore 119228, Singapore; 3 Department of Haematology-Oncology, National University Cancer Institute, Singapore 14 Medical Drive, #12-01, Singapore 117599, Singapore; 4 Department of Medicine, Yong Loo Lin School of Medicine, National University of Singapore, 1E, Kent Ridge Road, NUHS Tower Block Level 10, Singapore 119228, Singapore; 5 Discipline of Pharmacy & Experimental Pharmacology, School of Biomedical Sciences & Pharmacy, Room 607c, Medical Sciences Building, University of Newcastle, Callaghan, N.S.W, 2308, Australia

**Keywords:** Gastric cancer, Functional assessment of cancer therapy- gastric, Quality of life, Reliability, Validity, Chinese

## Abstract

**Background:**

Quality of life (QoL) assessment has become an important aspect of the clinical management of gastric cancer (GC), which poses a greater health threat in Chinese populations around the world. Functional Assessment of Cancer Therapy-Gastric Module (FACT-Ga), a questionnaire developed specifically to measure QoL of patients with GC, has never been validated in Chinese subjects. The current study was designed to examine the psychometric properties of FACT-Ga as a GC specific QoL instrument for its future use in Chinese populations.

**Methods:**

A sample of 67 Chinese patients with GC in the National University Hospital, Singapore was investigated cross-sectionally. The participants independently completed either English or Chinese versions of the FACT-Ga and the European Quality of Life-5 Dimensions (EQ-5D). Reliability was measured as the Cronbach’s α for EQ-5D, and five subscale scores and two total scores of FACT-Ga. The sensitivity to patients’ clinical status was evaluated by comparing EQ-5D and FACT-Ga scores between clinical subgroups classified by Clinical Stage and Treatment Intent. The construct validity of FACT-Ga was assessed internally by examining the item-to-scale correlations and externally by contrasting the FACT-Ga subscales with the EQ-5D domains.

**Results:**

For both FACT-Ga and EQ-5D, patients treated with curative intent rated their QoL higher than those treated for palliation, and early stage patients scored higher than those in the late stage. The sensitivity to clinical status of FACT-Ga scores were differential as four of seven FACT-Ga scores were significant for Treatment Intent while only one subscale score was significant for Clinical Stage. Six FACT-Ga scores had Cronbach’s α of 0.8 or above indicating excellent reliability. For construct validity, 45 of 46 items converged about their respective subscales. The monotrait-multimethod correlations between QoL constructs of FACT-Ga and EQ-5D were stronger than the multitrait-multimethod correlations as theoretically hypothesized, suggesting good convergent and discriminant validities.

**Conclusions:**

Given the excellent reliability and good construct validity, FACT-Ga scores are able to distinguish patient groups with different clinical characteristics in the expected direction. Therefore FACT-Ga can be used as a discriminative instrument for measuring QoL of Chinese patients with GC.

## Introduction

Quality of life (QoL) has been increasingly recognized as an important outcome for cancer therapy [1]. QoL assessment has special clinical significance in the management of gastric cancer (GC) patients, as the malignancy in a large proportion of GC patients is manifested in the form of ascites or lymphangitis carcinomatosa, thus rendering the ordinary response criteria such as tumor size less informative. A valid and reliable instrument is critical to obtain QoL data of both clinical and public health relevance [2].

The Functional Assessment of Chronic Illness Therapy (FACIT) is a collection of questionnaires developed primarily for the QoL measurement for various cancers. FACIT has been established internationally as one of the reliable and valid QoL measurement systems in clinical oncology [3]. The core module of FACIT, the Functional Assessment of Cancer Therapy (FACT-G), has advantage over other cancer-generic QoL instruments in sample size requirements [4]. Simply adding cancer specific symptom items for a particular organ to FACT-G derives an organ-specific cancer QoL instrument, such as those for colon, lung and breast cancer [3]. These instruments have been validated and widely used in different populations internationally [5-7].

However, the GC specific module based on FACT-G, the Functional Assessment of Cancer Therapy-Gastric (FACT-Ga) [8], has not been sufficiently validated. There are only two recent publications validating FACT-Ga in Western populations [9,10] and no data for Chinese populations, who have a higher incidence and mortality of GC [11,12] not limited to mainland China, but also in ethnic Chinese communities in other countries [13,14].

Previous attempts have been made to validate FACT-G for its use in cancer patients of Chinese ethnicity [15-17], however, GC was not covered explicitly in these studies. Therefore, how well the FACT-G or FACT-Ga performs in measuring the QoL of Chinese patients with GC remains unknown. In Singapore, Chinese constitutes 75% of the entire population and carries an intermediate risk of GC in general and a high risk in males aged 50 years or older [18]. Furthermore, the multilingual culture in Singapore enables the validation of both the English and Chinese versions of the instrument and its use in a broader population base. As such, we designed this study to examine the psychometric properties of FACT-Ga with a sample of GC patients from the Singapore Chinese population. Our aim was to validate FACT-Ga as a GC specific QoL instrument for its use in Chinese populations. Empirical evidence of the reliability, construct validity and sensitivity to patients’ clinical status of FACT-Ga was reported.

## Material and methods

### Study sample

The study was conducted between November 2010 and October 2011 at the National University Hospital (NUH), Singapore. Patients from the Surgery Clinic and the National University Cancer Institute at the NUH were recruited using the following inclusion criteria, 1) Chinese ethnicity, 2) age 45 years or older, 3) histologically confirmed GC, 4) at least two weeks after an operation, 5) no evidence of other concurrent severe medical conditions, and 6) able to complete the questionnaires independently. The study was approved by the Institutional Review Board at the NUH. All participants provided written informed consent.

### Quality of life instruments and data collection

Patients with GC were referred by their consulting doctors to the interviewer for an assessment of their eligibility for this study. Once a patient met the inclusion criteria, the interviewer would lead the patient to a separate room for the face-to-face session. In the presence of the interviewer, patients independently completed two questionnaires, the FACT-Ga (Version 4) and the 3-level European Quality of Life-5 Dimensions (EQ-5D) in original English or Chinese in accordance with their language preference. The order of the two instruments was randomized to rule out order effects [19].

FACT-Ga evaluates the participant’s QoL over the past seven days and consists of two parts: 1) the core module FACT-G which comprises four general subscales, namely physical well-being (PWB), social well-being (SWB), emotional well-being (EWB) and functional well-being (FWB), and 2) a 19-item gastric cancer subscale (GCS) surveying GC symptoms and adverse effects associated with GC treatment. The FACT-Ga items are rated on a 5-point Likert scale. Summation of item scores produces scores for the PWB, SWB, EWB, FWB and GCS subscales. The aggregate of the PWB, SWB, EWB and FWB scores is the FACT-G total score. The FACT-Ga total score is the sum of the FACT-G total score and GCS subscale scores.

For the EQ-5D, participants were required to rate their QoL on the day of interview. The EQ-5D questionnaire measures five domains of the patient’s life, i.e. Mobility, Self-Care, Usual Activities, Pain/Discomfort and Anxiety/Depression. The domain scores are used to compute a utility anchored between 0 (death) and 1 (full health) [20].

For the EQ-5D utility and FACT-Ga scores, high values indicate a better quality of life, while high EQ-5D domain scores indicate worse health status. Clinical information was collected directly from the patients’ case-notes.

### Statistical analysis

The English and Chinese questionnaires were pooled together for the analysis as the measurement equivalence between two language versions of EQ-5D and FACT-G has been previously confirmed in Singaporean Chinese [16,21]. As participants were given the option not to answer the seventh item of the SWB subscale, GS7 (*“I am satisfied with my sex life”)*, 32 (48%) participants did not respond to this item. The SWB subscale scores for these subjects were prorated following the FACIT Administration and Scoring Guidelines [22].

Reliability was quantified as internal consistency by using the Cronbach’s *α*. An alpha value equal to or greater than 0.70 was considered satisfactory [23]. The sensitivity to clinical severity was tested in relation to the clinical variables, Treatment Intent (curative vs. palliative) and Clinical Stage (6^th^ American Joint Committee on Cancer (AJCC) Stage 0,1,2,3 vs. AJCC Stage 4), using the effect size and the significance level of the Student’s *t*-test. Construct validity of FACT-Ga was first evaluated by examining the Pearson correlation coefficient between an item and its own scale after correction for overlap [24]. An item-to-scale correlation reaching a minimum of 0.4 was considered a good convergent validity for the scale.

The convergent and discriminant validities were further evaluated using the multitrait-multimethod (MTMM) approach for incomplete design, which explored all the inter-scale correlations among the EQ-5D domains and FACT-Ga subscales [25]. As EQ-5D is a generic QoL instrument and FACT-Ga is a GC specific QoL instrument, there is no one-to-one correspondence of the QoL constructs across two questionnaires. For the data interpretation, we hypothesized theoretically that the FACT-Ga PWB, EWB and FWB subscales corresponded to the EQ-5D Pain/Discomfort, Anxiety/Depression and Usual Activity domains respectively, whilst the FACT-Ga GCS subscale corresponded to the EQ-5D Pain/Discomfort domain. The correlations between these QoL construct pairs are the monotrait-multimethod correlations in the MTMM correlation matrix. As an incomplete design was adopted, these correlations would not line up as a validity diagonal. The remaining cross-instrument inter-scale correlations are termed multitrait-multimethod correlations. Both convergent validity and discriminant validity are supported if the strengths of the monotrait-multimethod correlations are stronger than the multitrait-multimethod correlations as expected *a priori*. The statistical software package SPSS v17 (SPSS Inc, Chicago, Illinois) was used to perform all analyses. A p-value less than 0.05 was considered statistically significant.

## Results

Of the 80 GC patients approached, 75 agreed to participate (94% response rate). Three participants were illiterate and five participants had severe comorbidities or substantial missing information and so were excluded from the study sample. Finally, a total of 67 consecutive GC patients were recruited into the study.

The demographic and clinical characteristics of the sample were summarized in Table 1. The mean age of the participants was 67 years and the average survival time was 2.13 years after diagnosis. Approximately 75% of participants chose the Chinese version of the questionnaires. Our sample had a representative range of clinical cases, including patients diagnosed with AJCC stage 0 to stage 4, those with or without previous surgery, metastases, and a history of chemo/radiotherapy. The majority of these patients received treatment in hospital with a curative (71.6%) rather than a palliative intent.

**Table 1:** Demographic and clinical characteristics of the sample

**Variables**	**Categories**	Value^*^
Age (years)		67.4 (11.9)
Survival time (years)		2.1 (2.5)
Gender	Male	43 (64.2)
	Female	24 (35.8)
Language Version	Chinese	50 (74.6)
	English	17 (25.4)
AJCC stage (6^th^ edition)	Stage 0	3 (4.5)
	Stage 1	19 (28.4)
	Stage 2	14 (20.9)
	Stage 3	13 (19.4)
	Stage 4	18 (26.9)
Metastasis	No	54 (80.6)
	Yes	13 (19.4)
Treatment Intent	Curative	48 (71.6)
	Palliative	19 (28.4)
History of surgery	Total gastrectomy	12 (17.9)
	Subtotal gastrectomy	37 (55.2)
	Surgical procedure	4 (6.0)
	No surgery	14 (20.9)
History of chemo/radiotherapy	No	44 (65.7)
	Yes	23 (34.3)

^*^Values are mean (standard deviation) for interval variables and number (percent) for categorical variables.

The distributions of the FACT-Ga scores and EQ-5D utility, the ceiling effect and the reliability index were presented in Table 2. Each score had a slightly left-skewed distribution as the medians were greater than the means. A floor effect was not observed but a ceiling effect was present for all scorers with notable values for the PWB (26.87%), SWB (16.42%), EWB (16.42%) subscales and the EQ-5D utility (47.76%) [26]. Cronbach’s α values showed excellent reliability for both FACT-Ga and EQ-5D, as only the EWB subscale had a value below 0.8 [23].

**Table 2:** Score distributions, ceiling effect, reliability and item-scale convergence of the FACT-Ga and EQ-5D

	**Mean (SD)**	**Median**	**Range**	Ceiling effect^*^(%)	**Cronbach’s α**	**Item correlation with its own scale (range)**	**No of success**^**†**^**/total (%)**
PWB	24.3 (4.08)	26	5–28	26.9	0.85	0.47–0.73	7/7 (100)
SWB^‡^	21.5 (5.45)	23	8–28	16.4	0.82	0.44–0.68	7/7 (100)
EWB	20.1 (3.40)	21	10–24	16.4	0.62	0.08–0.56	4/6 (67)
FWB	19.3 (6.58)	21	5–28	10.5	0.89	0.47–0.81	7/7 (100)
GCS	59.9 (12.18)	63	27–76	4.5	0.90	0.25–0.78	15/19 (79)
FACT-G	85.2 (14.55)	86	47–108	3.0	0.89	0.08–0.81	25/27 (93)
FACT-Ga	144.7 (24.51)	149	74–184	3.0	0.93	0.08–0.81	40/46 (87)
EQ-5D	0.80 (0.28)	0.88	−0.06–1	47.8	0.81	-	-

^*^Percentage of subjects who reached the highest score of the scale.

^†^Success refers to an item’s Pearson correlation coefficient with its own scales equal or greater than 0.4.

^‡^Cronbach’s α was computed based on 6 items exclusive of GS7.

In the analysis of sensitivity (Table 3), the FACT-Ga QoL measures corresponded well with clinical severity as indicated by Treatment Intent and Clinical Stage. Patients treated with curative intent rated their QoL higher than those treated for palliation. Patients diagnosed with an early stage of GC (AJCC stages 0, 1, 2, 3) scored higher than patients diagnosed with the late stage of the disease (AJCC stage 4). These observations were in line with the direction of the overall QoL measured by the EQ-5D utility, which was significantly different between the subgroups of both clinical variables. However, not all FACT-Ga scores showed statistical significance in the comparisons between clinical subgroups. The EWB and FWB subscales, FACT-G and FACT-Ga were significantly different between curative and palliative patients with a moderate effect size from 0.56 to 0.74 [27], while for the Clinical Stage, only the GCS subscale achieved statistical significance with an effect size of 0.64, and the SWB subscale achieved a borderline significance level of p = 0.079.

**Table 3:** Sensitivity of FACT-Ga scores and EQ-5D utility to clinical severity

	**Treatment intent**				Clinical stage^*^			
	**Curative (n = 48)**	**Palliative (n = 19)**	**Effect size**	**P**	**Early stage (n = 49)**	**Late stage (n = 18)**	**Effect size**	**P**
	**Mean** **±** **SD**	**Mean** **±** **SD**			**Mean** **±** **SD**	**Mean** **±** **SD**		
PWB	24.8 (3.90)	23.1 (4.36)	0.45	0.112	24.7 (4.01)	23.4 (4.24)	0.32	0.264
SWB	21.3 (5.86)	21.9 (4.34)	−0.10	0.686	20.9 (5.94)	23.1 (3.48)	−0.36	0.079
EWB	20.6 (2.95)	18.7 (4.11)	0.65	0.036	20.4 (2.97)	19.3 (4.38)	0.36	0.256
FWB	20.6 (6.26)	16.0 (6.31)	0.74	0.008	19.9 (6.52)	17.5 (6.60)	0.37	0.180
GCS	61.4 (11.59)	56.1 (13.1)	0.46	0.106	61.8 (10.95)	54.8 (14.10)	0.64	0.035
FACT-G	87.4 (13.77)	79.6 (15.30)	0.56	0.049	85.9 (14.57)	83.2 (14.70)	0.18	0.511
FACT-Ga	148.5 (22.76)	135.2 (6.77)	0.59	0.044	147.4 (22.99)	137.5 (27.70)	0.43	0.146
EQ-5D	0.86 (0.24)	0.65 (0.33)	0.89	0.016	0.84 (0.25)	0.68 (0.33)	0.66	0.031

^*^Early stage: AJCC stages 0–3; Late stage: AJCC stage 4.

The construct validity of FACT-Ga was first evaluated by examining the item-scale convergence for each item in Table 2. Forty (87%) of the total of 46 FACT-Ga items had Pearson correlation coefficients of 0.4 or greater with their own scales, indicating satisfactory convergent validity. The remaining 6 items, GE2 and GE6 of the EWB subscale, and items C2, HN1, C5 and E6 of the GCS, had correlation coefficients below 0.4. However, the correlation coefficients of 5 items (GE6, C2, HN1, C5 and E6) had 95% confidence intervals (CI) covering 0.4 [24]. The last item GE2 (*“I am satisfied with how I am coping with my illness”)* had a correlation coefficient of 0.08 with its own subscale EWB, which was significantly smaller (*P =* 0.04) than its correlation with the FWB subscale (*r =* 0.43), fulfilling the definition of a definite scaling error [28].

The construct validity of FACT-Ga was further evaluated in the MTMM analysis using EQ-5D (Table 4). Basically, the overall QoL quantified as the EQ-5D utility and as the FACT-Ga or FACT-G total score were strongly correlated (*r =* 0.70 and *r =* 0.66 respectively). The MTMM correlation matrix described in detail the inter-scale correlation patterns within either instrument, and more importantly across different instruments. The latter correlations connecting the EQ-5D domains with the FACT-Ga subscales were summarized in the lower-left square of the MTMM matrix called Heteromethod Block. As hypothesised *a priori*, the four monotrait-multimethod correlations highlighted in Table 4 were generally higher than the multitrait-multimethod correlations. The correlation coefficients of the PWB subscale with the Pain/Discomfort domain (*r = −*0.66) and the EWB subscale with the Anxiety/Depression domain (*r = −*0.57) were the highest in their respective columns and rows. The correlation coefficient of GCS with the Pain/Discomfort domain (*r = −*0.63) was comparable to that of the PWB subscale. The correlation between the functional QoL constructs, the FWB subscale in FACT-Ga and the Usual Activity domain in EQ-5D was −0.49. Despite being the strongest correlation in the Usual Activity domain column, it was weaker than the two multitrait-multimethod correlations: the Anxiety/Depression domain with the FWB subscale (*r = −*0.52) and the Mobility domain with the FWB subscale (*r = −*0.58). As the FACT-Ga SWB subscale and EQ-5D Self-care domain were not conceptually related to any QoL construct of the other instrument, the correlations involving these two QoL constructs were the lowest of the respective rows or columns.

**Table 4:** Multitrait-multimethod correlations matrix between EQ-5D domains and FACT-Ga subscales

	**EQ-5D**					**FACT-Ga**				
	**Pain/Discomfort**	**Anxiety/Depression**	**Usual activities**	**Mobility**	**Self-care**	**PWB**	**EWB**	**FWB**	**GCS**	**SWB**
EQ-5D										
Pain/Discomfort	1									
Anxiety/Depression	0.52^**^	1								
Usual Activities	0.27^*^	0.37^**^	1							
Mobility	0.31^*^	0.44^**^	0.75^**^	1						
Self-care	0.25^*^	0.35^**^	0.66^**^	0.70^**^	1					
FACT-Ga										
PWB	−0.66^**^	−0.46^**^	−0.42^**^	−0.44^**^	−0.29^*^	1				
EWB	−0.46^**^	−0.57^**^	−0.48^**^	−0.45^**^	−0.25^*^	0.64^**^	1			
FWB	−0.39^**^	−0.52^**^	−0.49^**^	−0.58^**^	−0.45^**^	0.48^**^	0.60^**^	1		
GCS	−0.63^**^	−0.43^**^	−0.4^**^	−0.38^**^	−0.33^**^	0.80^**^	0.62^**^	0.48^**^	1	
SWB	−0.14	−0.38^*^	−0.20	−0.18	−0.20	0.13	0.30^*^	0.32^**^	0.13	1

Monotrait- multimethod correlations are highlighted in bold.

^*^ P < 0.05, ^**^p < 0.01.

## Discussion

Our study validated FACT-Ga in a clinically heterogeneous sample of Singaporean Chinese patients with GC. The study sample covered the full spectrum of clinical cases which would allow for applying the validated questionnaire to various diagnostic groups. To the best of our knowledge, this is the first study dedicated to validating FACT-Ga for the Chinese as the target population. Given that the target population lives in a bilingual culture, both English and Chinese versions of the questionnaires were validated so that the FACT-Ga would be applicable in mainland Chinese as well as overseas Chinese populations.

For this sample of outpatients, the measurement ability of the FACT-Ga appears to be limited in evaluating the QoL outcomes of patients who survived GC relatively well. The ceiling effect was observed for all scores, especially the PWB, SWB and EWB subscales, for which the percentage of patients rating themselves in perfect health exceeded a notable value of 15% (Table 2). The core module FACT-G also showed a ceiling effect when applied to other types of cancer patients from the same study population [17]. A ceiling effect above 15% could have a negative impact on other psychometric properties of an instrument [29], for example, the sensitivity to change, as supported by the finding that FACT-G is weak in detecting the improvement in a patients’ health status [9,17]. The evidence thus far seems to suggest that the existence of a ceiling effect of FACT-Ga compromises its potential as an evaluative QoL instrument.

The FACT-Ga questionnaire showed sensitivity to the clinical characteristics of different patients groups, supporting FACT-Ga as a discriminative QoL instrument. Treatment Intent and Clinical Stage are important concerns for doctors to consider when making a clinical decision. The QoL profile described by the FACT-Ga scores corresponded very well to the clinical classification by the two variables (Table 3). The patients in a more severe situation, i.e., those treated for palliation or those with the disease in the advanced stage rated their life worse. For either overall or specific QoL aspects, it is clear that the group differences are in the direction theoretically hypothesized and consistent with the findings from the EQ-5D.

Over and above its reflection of clinical severity of GC malignancy, the FACT-Ga scores exhibited differential sensitivity to clinical status. As suggested by the varying degree of the effect size and significance level of the *t*-test for each FACT-Ga score, the EWB and FWB subscales were sensitive to Treatment Intent, while the GCS and potentially the SWB subscales were sensitive to the patient’s clinical stage. The effects of clinical classifications are moderate on the QoL outcomes (Table 3). Furthermore, the FACT-Ga instrument revealed an interesting finding about the social aspect of the patient’s life, which was not measured in EQ-5D. The FACT-Ga SWB subscale scores were higher for severe cases than for less severe cases for both Treatment Intent and Clinical Stage. This direction was opposite to that demonstrated by other scales. It would be too simplistic to ascribe the finding to random variation. We speculated instead that the Chinese culture played a part in this observation considering that our study population was Chinese. Sympathy is the essence of Chinese value systems and it could naturally be inferred that severe GC patients would receive more love and care from the people close to them. The SWB subscale is a measure of a patient’s self-perception of family support and emotional closeness to friends. Therefore, the reverse trend of SWB scores is not unexpected and possibly culturally-specific.

In our study, the FACT-Ga instrument demonstrated excellent reliability in measuring the QoL of Chinese GC patients. Except for the EWB subscale, Cronbach's *α* values indicating internal consistency reliability were greater than 0.80 for the instrument and other subscales [23]. The EWB subscale had a Cronbach’s *α* of 0.62, comparable to 0.60 as previously reported [9], yet below the generally accepted standard of 0.70 [23]. As the Cronbach’s *α* of a scale is computed based on the inter-correlations among its constituent items, the extremely low item-to-scale correlation of the EWB subscale with its item GE2 (*r =* 0.08) was supposed to account for the suboptimal reliability of the EWB subscale [28]. After excluding item GE2, the EWB subscale had an improved Cronbach’s *α* of 0.72.

The Cronbach’s α of the SWB subscale was reported as 0.82, indicating excellent reliability of the SWB subscale, based on the 67 participants who completed the first six items of the SWB subscale. The seventh item of the SWB subscale, GS7 asking about the sex life of the patient, introduced a non-response rate of 48% (n = 32), which greatly reduced the sample size for computing the reliability index. The Cronbach’s α would drop to 0.77 based on the 35 participants with complete information for seven SWB items. Non-response to the item GS7 was common in FACT-G validation studies in different cancer populations [30,31]. Excluding GS7 in the calculation of Cronbach’s α for the SWB subscale has been practiced to minimize the detriment of missing information [30]. Doing so also prevented an unstable estimate of the reliability index due to an insufficient sample size, which was demonstrated by the Cronbach’s *α* varying from 0.26 to 0.86 in five small samples (n = 15) validating the FACT-Ga [10].

Construct validity of FACT-Ga was explored internally by examining the item-to-scale correlations for each item and externally by contrasting with EQ-5D. As hypothesized *a priori,* most items converged around their individual master subscales as required for good convergent validity. However, the Pearson correlation coefficient of item GE2 with the EWB subscale was only 0.08 with a 95% CI below 0.4. It was also associated with a definite scaling error implying that item GE2 should be included in the FWB subscale rather than the EWB subscale when FACT-Ga is used in Chinese GC patients [28]. This finding supports the results of previous cross-cultural studies investigating the factor structure of the FACT-G in Asian populations [15,32].

External validation involves a MTMM correlation matrix correlating the FACT-Ga subscales with the EQ-5D domains. The similar QoL constructs which were specified separately for EQ-5D and FACT-Ga yielded stronger monotrait-multimethod correlations than the multitrait-multimethod correlations in the Heteromethod block (Table 4). As shown by the monotrait-multimethod correlation coefficients, the PWB, EWB and GCS subscales are measuring the QoL aspects as intended. They are also able to discriminate the different aspects of a patient’s life [25]. With regard to a patient’s functionality, the FACT-Ga FWB subscale was not strongly related to the Usual Activity domain of EQ-5D as we hypothesized, but to two EQ-5D domains, the Mobility and Anxiety/Depression domains, with similar strengths of correlation (*r* = −0.58 and *r* = 0.52 respectively). This may reflect the fact that the SWB subscale score is an integration of the mental and physical functions of a patient’s life. The QoL constructs which are covered by only one questionnaire had the lowest cross-instrument correlations, for example, the SWB subscale of FACT-Ga and the Self-care domain of EQ-5D. These results confirmed and substantiated the convergent and discriminant validities of FACT-Ga.

However, several limitations in this study must be noted. We were only able to recruit outpatients who usually have a better current QoL and prognosis than those hospitalized for radical treatments or bedridden at home. We acknowledge that the validity and reliability estimates were influenced by the narrow sampling due to logistical difficulties [33]. However, considering the statistical property of Cronbach’s *α* and the correlations indicating construct validity, a more heterogeneous study sample generated by a wider patient pool would strengthen the current estimates [24]. With a cross-sectional sample, we were unable to assess test-retest reliability and the responsiveness of FACT-Ga measures to QoL change over time. Finally, the sample size is sufficient, yet modest, for a study validating a cancer specific questionnaire. This may explain in part why some t-tests comparing subscale scores were insignificant (Table 3). A bigger cohort with follow-up information would be necessary to consolidate the current findings.

## Conclusion

Our study demonstrated that, when used in a Chinese population, FACT-Ga is able to detect the group-differences in QoL outcomes between the clinically distinct patient groups. The total and subscale scores from FACT-Ga can be considered reliable and valid measures of the QoL of Chinese patients with GC. This evidence supports the use of FACT-Ga as a discriminative QoL instrument alone or as a supplement to a generic QoL instrument in clinical trials and routine clinical practice.

## Abbreviations

QoL: Quality of life; GC: Gastric cancer; FACIT: Functional assessment of chronic illness therapy; FACT-G: Functional assessment of cancer therapy; FACT-Ga: Functional assessment of cancer therapy- gastric; PWB: Physical well-being; SWB: Social well-being; EWB: Emotional well-being; FWB: Functional well-being; GCS: Gastric cancer subscale; NUH: National university hospital; EQ-5D: European quality of life-5 Dimensions; AJCC: American joint committee on cancer; MTMM: Multitrait-multimethod.

## Competing interests

The author(s) declare that they have no competing interests.

## Authors’ contributions

HJZ designed the study, collected and analyzed the data and drafted the manuscript. JBYS and WPY consulted and referred the patients, NL instructed the study design and data analysis, NN and SCL critically revised the draft, FZ and KGY oversaw all stages of research, KGY approved the draft for submission. All authors read and approved the final manuscript.

## Authors’ information

KGY is Dean of Yong Loo Lin School of Medicine and also the principal investigator of the Gastric Cancer Epidemiology, Clinical and Genetic Programme (GCEP), a hospital-based prospective study in which 4000 subjects at high risk of GC were recruited and followed for five years for the occurrence of GC. One of the objectives of GCEP is to develop a cost-effective screening algorithm in Singaporean Chinese. This study is nested within GCEP to provide the QoL data for the future cost-effectiveness analysis. Furthermore, it will facilitate the health outcome studies in GC.
